# Antigen Loss Variants: Catching Hold of Escaping Foes

**DOI:** 10.3389/fimmu.2017.00175

**Published:** 2017-02-24

**Authors:** Maulik Vyas, Rolf Müller, Elke Pogge von Strandmann

**Affiliations:** ^1^Experimental Tumor Research, Center for Tumor Biology and Immunology, Clinic for Hematology, Oncology and Immunology, Philipps University, Marburg, Germany; ^2^Institute of Molecular Biology and Tumor Research, Center for Tumor Biology and Immunology, Philipps University, Marburg, Germany

**Keywords:** antigen loss, leukemia, NK cells, T cells, dual-targeting immunoligand

## Abstract

Since mid-1990s, the field of cancer immunotherapy has seen steady growth and selected immunotherapies are now a routine and preferred therapeutic option of certain malignancies. Both active and passive cancer immunotherapies exploit the fact that tumor cells express specific antigens on the cell surface, thereby mounting an immune response specifically against malignant cells. It is well established that cancer cells typically lose surface antigens following natural or therapy-induced selective pressure and these antigen-loss variants are often the population that causes therapy-resistant relapse. CD19 and CD20 antigen loss in acute lymphocytic leukemia and chronic lymphocytic leukemia, respectively, and lineage switching in leukemia associated with mixed lineage leukemia (MLL) gene rearrangements are well-documented evidences in this regard. Although increasing number of novel immunotherapies are being developed, majority of these do not address the control of antigen loss variants. Here, we review the occurrence of antigen loss variants in leukemia and discuss the therapeutic strategies to tackle the same. We also present an approach of dual-targeting immunoligand effectively retargeting NK cells against antigen loss variants in MLL-associated leukemia. Novel immunotherapies simultaneously targeting more than one tumor antigen certainly hold promise to completely eradicate tumor and prevent therapy-resistant relapses.

## Introduction

In what is known as cancer immunoediting, the immune system not only tries to eradicate the evolving tumor but, in doing so, also shapes the immunogenicity of the tumor that may escape the immune control (1). Ultimately, the tumor cells that progress despite the immunosurveillance consist of one or more clones with lower visibility and/or higher resistance to the immune cells (1). For example, tumors often decrease the expression of components required for antigen presentation (MHC) and/or T cell activation (costimulatory molecules) as well as ligands for the NK cell-activating receptors in order to hide from the T and NK cells, respectively (1–4). Alternatively, tumor cells express ligands, which, upon binding to the respective checkpoint receptors such as CTLA-4 and PD-1 on T cells and KIR and CD94/NKG2A on NK cells, suppress their effector functions (5–9). The following sections review the current targeted therapies and the evidences of relapses associated with antigen loss variants in leukemia. Several therapeutic approaches including a dual-targeting immunoligand to manage this challenging clinical scenario are discussed.

## Targeted Immunotherapies—Current Status in Leukemia

Acute leukemia represents an uncontrolled proliferation of the immature immune precursor cells and are further classified based on the lineage of the affected immune cell. Acute lymphocytic leukemia (ALL) affects the cells from the lymphoid lineage in contrast to the leukemia of myeloid cells, collectively known as acute myeloid leukemia (AML) (10). Both lymphoid and myeloid leukemia exploit the abovementioned and several other immune evasive strategies [reviewed in Ref. (6)]. However, the fact that tumor cells have to evade the immune system in order to be clinically relevant disease also supports the idea that immune system, when properly activated, can fight the cancer.

Over the last three decades, the cancer immunotherapy field has seen much progress and most of its success can be attributed to the targeted therapies against leukemia (11). The most promising immunotherapeutic options for leukemia include targeted approaches such as chimeric antigen receptor (CAR) modified T cells (CAR-T cells) and antibody-based therapies that activate T and NK cells (11). Within the CAR construct, extracellular antibody-derived scFv confers the antigen specificity, while the intracellular signaling domains (from T cell receptor and costimulatory molecule) provide the activation signal to the engineered T cells (12). Various CAR-T cells have entered the clinical studies for leukemia and the most advanced CAR is against CD19, which is being tested for ALL (13, 14). Blinatumomab, a bispecific T cell engager against CD19 and CD3 that recently got the FDA approval for the treatment of ALL, is an antibody-based molecule that also activates T cells, albeit via CD3, against the CD19-bearing target cells (15). NK cells, like T cells, have equally contributed to the clinical success of cancer immunotherapy against leukemia. For example, NK cells serve as an important effector population in chronic lymphocytic leukemia (CLL) patients who mediate antibody-dependent cell-mediated cytotoxicity through FcγRIIIa (CD16a) receptor engagement by the FDA-approved anti-CD20 antibodies (rituximab, obinutuzumab, and ofatumumab), anti-CD52 antibody alemtuzumab, and other promising anti-CD19 antibodies (MEDI-551 and XmAb5574) that are currently in clinical trials (16). In addition to the conventional antibodies, there are numerous novel approaches currently in preclinical development that aim to harness NK cell activity against cancer [reviewed in Ref. (8)].

Although many of the targeted immunotherapies have produced unprecedented responses in leukemia, especially in chemorefractory patients, the complete remissions observed following such therapies are not long-lasting and a large variety of leukemia cases are presented with relapses that are aggressive and difficult to manage. This dismal scenario emphasizes the intratumoral heterogeneity that is driven by the intrinsic factors such as accumulation of genetic and epigenetic mutations during tumor progression and extrinsic factors imposed by therapeutic pressure and tumor microenvironment (17, 18).

## Occurrence of Antigen Loss Variants in Leukemia

Around 30% of acute leukemia patients experience a relapse with occasional co-presentation of a phenomenon known as “lineage switch.” Lineage switching occurs when acute leukemia that was initially classified as lymphoid or myeloid subtype according to the standard French–American–British guidelines shows opposite lineage when relapsed (10, 19). This phenomenon is often associated with poor prognosis and therapy resistance regardless of whether it emerged due to the lineage conversion of the original malignant clone or the selective outgrowth of a new leukemic clone (10). Out of the two possibilities, lymphoid to myeloid lineage switch is more frequently observed with more cases reported in children and often associated with the mixed lineage leukemia (MLL) gene rearrangements on chromosome 11q23 (20, 21).

Most cases of lineage switch have been reported in patients who had undergone some sort of targeted therapy. CD19-targeting immunotherapies including a bispecific antibody blinatumomab and CAR-expressing T cells have been very effective in chemorefractory B cell ALL. Anti-CD19/CD3 antibody blinatumomab redirects endogenous T cells in patients (15), while anti-CD19 CAR T cells are genetically engineered to be specifically activated against CD19 expressing target cells when infused in patients (22). Despite exceptional responses associated with these targeted therapies, some patients relapse and in many cases loss of CD19 antigen is reported. Duffner et al. reported a patient who was diagnosed with B-ALL associated with MLL-gene rearrangements but with no evidence of mixed lineage phenotype. Although blinatumomab therapy led to the complete disappearance of leukemic B cells, the patient relapsed with a more aggressive monocytic AML, which was negative for typical lymphoid markers such as CD19 (20). Similarly, CD19-specific CAR-T cell therapy could achieve complete response in all seven MLL-rearranged B-ALL patients. However, two of the seven patients relapsed with clonally related AML with no expression of B lymphoid antigens (21). Interestingly, both patients who showed lineage switch also had the presentation of cytokine release syndrome (21). Interleukin-6 (IL6), a key mediator of cytokine release syndrome, has also been shown to induce lymphoid to myeloid dedifferentiation *in vitro* (23) and *in vivo* (24). Although this is an indication of myeloid dedifferentiation of the original lymphoid blasts as an indirect effect of CAR-T cell therapy, it is also possible that myeloid clone is already present along with the lymphoid blasts, albeit below detection level, and is selected following the lymphoid-directed therapy. Ruella et al. recently described the presence of a small CD19-negative population in B-ALL patients before the administration of anti-CD19 CAR-T cell therapy (CTL019). Although there were no cases involving lineage switch, patients relapsed with CD19-negative B cell tumor following the CAR-T cell therapy (CTL019) and, as proposed by the authors, was most likely due to the selective outgrowth of the original CD19-negative subclone (13, 25). Beyond targeted immunotherapies, the phenomenon of lineage switch has also been observed following chemotherapy. As reported by Park et al., four patients of childhood B cell lineage ALL were treated with chemotherapy and were later presented with the relapse of clonally related AML (one patient) or a novel AML clone (three patients) (19).

While the link between treatment and lineage switching is not clear, the precise mechanism of antigen loss following mAb therapy is identified in several B cell malignancies. Rituximab, a chimeric antibody against CD20, has become a standard therapeutic option for various B cell (CD20^+^) malignancies including non-Hodgkin lymphoma (NHL), follicular lymphoma, diffuse large B cell lymphoma, and CLL (26, 27). The loss of CD20 antigen following rituximab therapy has been observed for follicular lymphoma (27), B cell NHL (28), and CLL (29). Two main mechanisms have been reported for CD20 loss from the CLL cells following rituximab (anti-CD20 mAb) treatment. While CD20 internalization by malignant B cells plays a minor role, the majority of CD20, along with the bound rituximab, is removed by the Fcγ receptor-expressing monocytes and macrophages in a process called as trogocytosis or shaving (30–32). This does not only result in the rapid clearance of rituximab following the infusion but also leads to selection of CD20-negative CLL cells that are resistant to anti-CD20 therapy. Similarly, CD19 internalization is also reported by anti-CD19 antibody XmAb5574 in CLL (33). Interestingly, Jones et al. reported the loss of CD19 from the CLL cells during the shaving (trogocytosis) of anti-CD20 rituximab. It was shown that CD19 was also transferred from B cells to monocytes in Fc receptor-dependent manner (34). Moreover, antigen loss in CLL is not only associated with the mAb therapy, for example, decrease in the cell surface expression of CD20 is observed by an immune modulating agent lenalidomide (26) or following the long-term *in vitro* coculture with mesenchymal stromal cells (29).

## Therapeutic Strategies to Combat the Antigen Loss Variants

As most tumor relapses involving antigen loss have been observed following antigen-specific therapies, one plausible solution is to use therapeutic approaches that are more general in their specificity and do not depend upon a particular tumor antigen. Immunotherapy with cytokine(s) such as IL2, IL12, and IL15 act via enhancing NK and T cell-mediated immune response against tumor (35). Although side effects associated with cytokines (e.g., IL2 and interferons) greatly limit their current use in the clinics, this approach still holds promise especially at lower doses and in combination with other anti-cancer therapies (35). Alternatively, checkpoint blockade involves blocking of the inhibitory receptors on immune cells to reverse the immune suppression by tumor cells (36, 37). Recent success in blocking of inhibitory receptors on T cells such as CTLA-4 and PD-1 by FDA-approved antibodies (checkpoint inhibitors) has led to the development of novel checkpoint inhibitors blocking NK cell inhibitory receptors KIR (lirilumab, Innate Pharma) and CD94/NKG2A (IPH2201, Innate Pharma) (36–38). The advantage is that such immune-modulatory approaches aim to promote an overall antitumor environment and are predicted to be less susceptible to the limitations associated with tumor heterogeneity and antigen loss (39). However, treatment options with no specificity for tumor are less likely to be curative as mono-agents and are often associated with the systemic side effects as observed in the form of immune-related adverse events following the checkpoint blockade approach (40).

Another strategy is to broaden the specificity of the current targeted therapies that have already shown promise in the clinics. CAR-T cells with dual specificities have been developed to improve T cell targeting of tumor cells even when one of the antigens is lost from the cell surface. A prototype CAR T cell with two distinct antigen-specific scFvs in tandem (TanCAR) retained T cell activity against antigen loss variants (41). The treatment of B-ALL patients enrolled in the pediatric CTL019 trial (the University of Pennsylvania/Children’s Hospital of Philadelphia) with CD19-specific CAR-T cells led to the outgrowth of CD19-negative malignant clone, which retained the expression of an IL3 receptor α chain (CD123) (25). Taking advantage of this, Ruella et al. developed CD19/CD123 CAR-T cells and proved its ability to completely eradicate the primary B-ALL blasts (CD19^+^CD123^+^ and CD19^-^CD123^+^) and to prevent the CD19 antigen loss relapse in an immunodeficient (NSG) mouse model (25). Despite the encouraging progress with the dual-specific CAR-T cell approach, major safety concerns typically associated with CAR-T cell therapy such as “on-target, off-tumor toxicity” and “cytokine release syndrome” would demand an equal attention (42).

Alternatively, NK cells, unlike T cells, express a diverse array of activating and inhibitory receptors to sense for the presence of stressed, virally infected or malignant cells. Moreover, there are multiple ligands for some of the activating receptors on NK cells (43). For example, the natural killer group 2 member D (NKG2D), an activating receptor on NK cells, can induce NK cell effector functions upon binding to any of the natural ligands such as UL16-binding proteins (ULBP1-6) and MHC-I-related chains (MICA/B) (43). This makes NK cells unlikely to succumb to the tumor heterogeneity and antigen loss provided that malignant cells remain visible to the NK cell scanning. However, the ligands for the NK cell-activating receptors, including NKG2D, are occasionally lost from the surface of leukemic cells in order to evade NK cell immunity (2, 3, 44). Of note, as shown by the recent work of Deng et al., soluble MULT1, a murine NKG2D ligand, played an indirect role in promoting NK cell immunity suggesting that soluble ligands may be more than inhibitory for overall NK cell activity (45). Our group has developed a therapeutic strategy to resensitize leukemic cells for NKG2D-dependent NK cell attack. To this end, we have developed and tested several bi- and trispecific recombinant immunoligands containing an NKG2D ligand ULBP2 fused to the various tumor antigen-specific scFvs (46–48). The idea is that these immunoligands will bind specifically to the tumor antigens and will coat the tumor cells with ULBP2 ligand. This will turn the otherwise NK cell-resistant tumor cells visible to NK cells for the attack. This was recently tested for the trispecific immunoligands (triplebodies) against CLL and MLL cells, which showed successful NK cell-mediated killing of leukemic cells in both, *in vitro* and *in vivo* settings (47).

**Figure 1:**
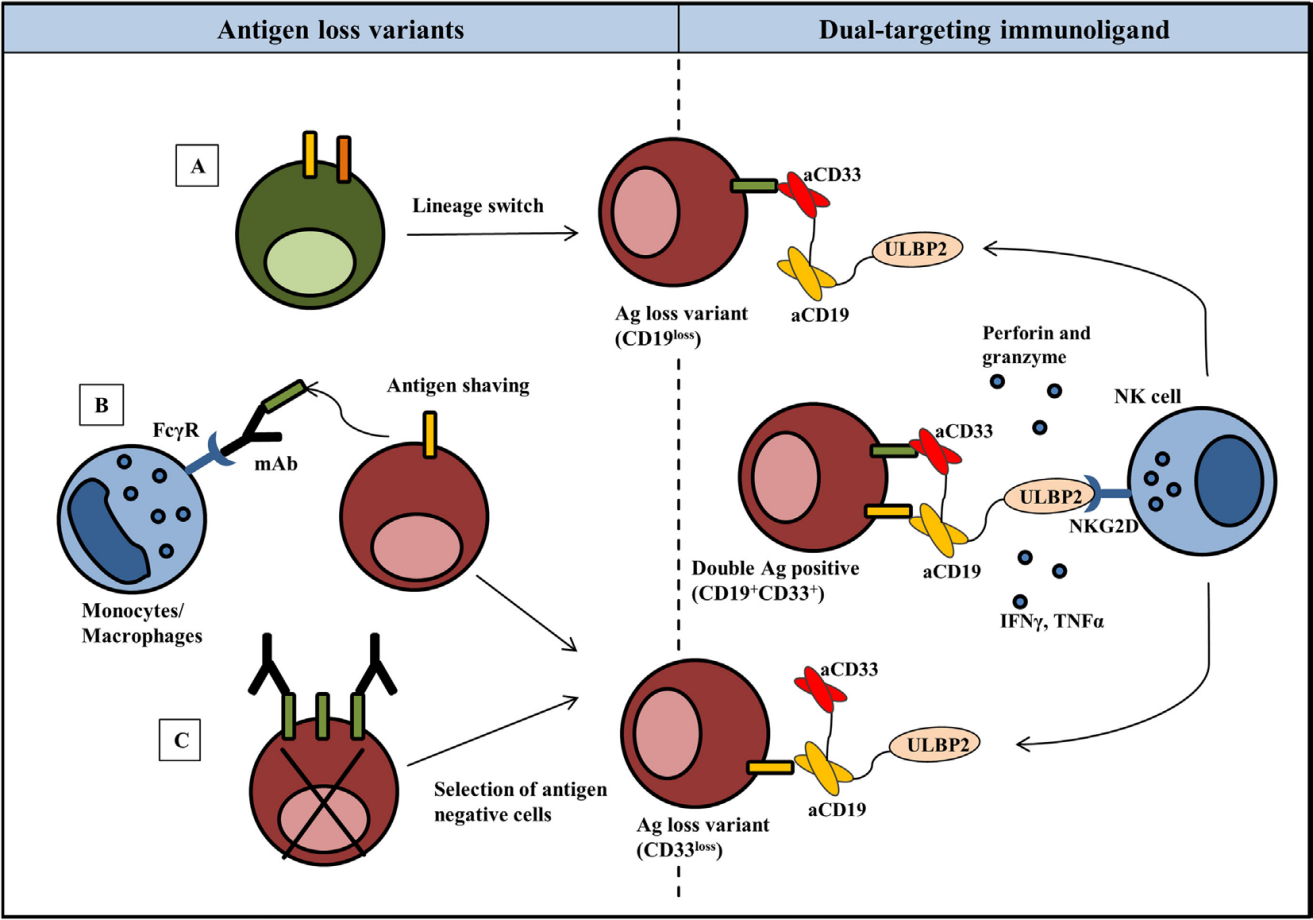
**Harnessing NK cells to control antigen loss variants: rational for the dual-targeting immunoligand approach**. Emergence of antigen loss variants in most cases is seen following targeted therapy and can be associated with lineage switching **(A)**, shaving or trogocytosis of antigen–antibody complexes from the tumor cells **(B)** or selective outgrowth of antigen-negative cells **(C)**. NK cell activating dual targeting immunoligand (triplebody) consists of two scFvs against distinct antigens on tumor cells and a natural ligand to activate NK cells. As an example, ULBP2-aCD19-aCD33 (dual targeting triplebody) binds not only to the double antigen-positive (CD19^+^CD33^+^) target cells but also to the antigen loss variants. ULBP2, now coated on the target cells, activates NK cell effector functions via NKG2D receptor resulting in the killing of tumor cells by perforin and granzymes and secretion of IFNγ and TNFα. For simplicity, cross-linking is only shown between CD19^+^CD33^+^ target cells and NK cell; however, identical NK cell targeting is possible in response to antigen loss variants.

**Figure 2:**
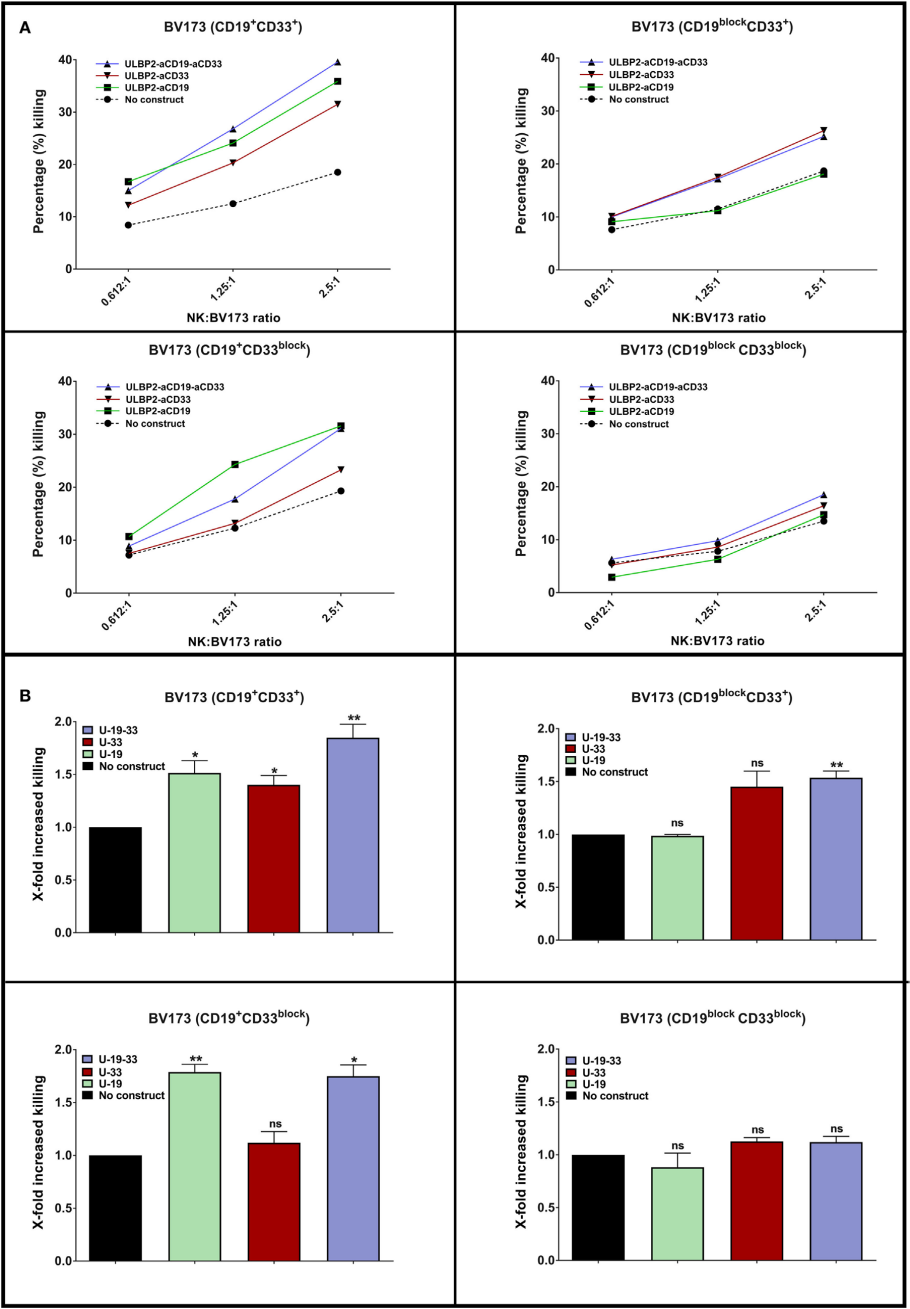
**A dual targeting triplebody ULBP2-aCD19-aCD33 mediates NK cell-dependent killing of antigen loss variants**. **(A)** NK cells were purified from healthy donor by negative selection and were primed by IL2 (200 U/ml) + IL15 (10 ng/ml) cytokines for 15–18 h (overnight). Next day, primed NK cells were incubated with DiR dye-labeled BV173 cells at indicated effector to target (E:T) ratio for 3 h. The incubation was continued either alone (No construct) or in the presence of 100 nM of immunoligand (U-19: ULBP2-aCD19, U-33: ULBP2-aCD33, U-19-33: ULBP2-aCD19-aCD33). After incubation, 7-AAD was added and 7-AAD-positive cells within DiR-positive gate indicated dead BV173 cells. One representative toxicity assay is shown. **(B)** Cumulative analysis of four independent toxicity assays at 2.5:1 (E:T) ratio (*N* = 4; each *N* represents an independent healthy NK cell donor). Error bars indicate SEM and statistical analysis by one-way ANOVA.

The ability of a dual-targeting triplebody ULBP2-aCD19-aCD33 to target antigen loss variants is showed in the present report. The term “dual-targeting triplebody” represents a trispecific immunoligand targeting two distinct antigens such as CD19 and CD33 in the case of ULBP2-aCD19-aCD33 against a B-cell precursor leukemic cell line BV173. The rational of this approach is that ULBP2-aCD19-aCD33 would coat not only the CD19- and CD33-positive target cells such as leukemic cells with MLL phenotype but also any existing or newly emerging clones that lost one of the antigens (Figure 1). ULBP2-aCD19 and ULBP2-aCD33, the bispecific immunoligands either targeting CD19 or CD33, would fail in this regard. To mimic the antigen loss variants of BV173 cell line, CD19 and/or CD33 antigens were preblocked using molar excess of CD19- or CD33-specific scFv moieties (aCD19scFv or aCD33scFv) that lacked an ULBP2 ligand. This has previously shown to completely abolish binding of the immunoligands and subsequent killing of target cells in an antigen-specific manner (47). As shown in Figure 2, when both antigens were accessible on BV173 (CD19^+^CD33^+^), all three immunoligands significantly enhanced the NK-cell-dependent killing of BV173 cells, albeit depending upon the expression level of the respective antigen. Of note, the surface expression of CD19 on the BV173 is several fold higher compared to CD33 (47, 49). When CD19 antigen was blocked (CD19^block^CD33^+^) by preincubation with aCD19scFv construct, BV173 killing induced by ULBP2-aCD19 was completely abolished while ULBP2-aCD33 and ULBP2-aCD19-aCD33 retained their toxic effects. Similarly, CD33 blocking on BV173 (CD19^+^CD33^block^) by aCD33scFv could abolish killing by ULBP2-aCD33 but not by ULBP2-aCD19 and the triplebody. Only, simultaneous blocking of both CD19 and CD33 antigens could abolish the killing induced by the dual-targeting triplebody ULBP2-aCD19-aCD33. This prototype immunoligand can also be modified to target a different combination of antigens such as CD19 and CD20 in case of CLL. Theoretically, it is also possible that tumor clones that have lost the expression of both antigens preexist within the heterogeneous tumor population and can be further selected even after dual-targeting approach. Moreover, this is also relevant in the context of antigen loss following targeted therapy as simultaneous loss of CD19 and CD20 antigens has been noted following rituximab therapy (34). Therefore, clinical success of the dual-targeting strategy will require careful selection of the tumor antigen pair and combination therapies should be considered in the case of double antigen loss.

Although this study focused on NK cell-dependent effects, NKG2D is also a shared activating receptor on γ/δ T cells and a coactivating receptor on CD8^+^ T cells. NKG2D-dependent antitumor effector functions of both of these T cell populations have been reported by us and others. Therefore, we believe that NKG2D targeting would facilitate a more dynamic immune reaction involving both, innate and adaptive arms. In human and mice, chronic stimulation of NKG2D receptor by membrane bound ligands leads to the reduced surface expression of NKG2D receptor (50, 51). However, ULBP2 is not as effective as MICA in causing downmodulation of NKG2D receptor (51), and we do not anticipate that the recombinant protein will be retained in the body fluids for a relevant period to cause significant downmodulation of NKG2D receptor.

Taken together, incorporating additional tumor specificity to the current mono-targeting T and NK cell-based therapies appears to be a promising approach to prevent or treat antigen loss relapse. Their ultimate clinical benefits may be more accurately predicted by addressing whether there are any additional adverse effects that are particularly associated with dual specificities.

## Ethics Statement

The collection of and the experiments with human NK cells from healthy volunteers were approved by the local ethics committee of the University of Cologne under reference number 11-140. Donors provided written consent in accordance with the Declaration of Helsinki.

## Author Contributions

MV and ES contributed to the design and analysis of the experiment. MV performed the experiments. MV, RM, and ES participated in writing and reviewing of the manuscript.

## Conflict of Interest Statement

The authors declare that the research was conducted in the absence of any commercial or financial relationships that could be construed as a potential conflict of interest.

## References

[1] Dunn GP Bruce AT Ikeda H Old LJ Schreiber RD. Cancer immunoediting: from immunosurveillance to tumor escape. Nat Immunol (2002) 3(11):991–8. 10.1038/ni1102-991 12407406

[2] Reiners KS Topolar D Henke A Simhadri VR Kessler J Sauer M Soluble ligands for NK cell receptors promote evasion of chronic lymphocytic leukemia cells from NK cell anti-tumor activity. Blood (2013) 121(18):3658–65. 10.1182/blood-2013-01-476606 23509156 PMC3643764

[3] Nuckel H Switala M Sellmann L Horn PA Durig J Duhrsen U The prognostic significance of soluble NKG2D ligands in B-cell chronic lymphocytic leukemia. Leukemia (2010) 24(6):1152–9. 10.1038/leu.2010.74 20428196

[4] Salih HR Holdenrieder S Steinle A. Soluble NKG2D ligands: prevalence, release, and functional impact. Front Biosci (2008) 13:3448–56. 10.2741/2939 18508446

[5] Yang H Bueso-Ramos C DiNardo C Estecio MR Davanlou M Geng QR Expression of PD-L1, PD-L2, PD-1 and CTLA4 in myelodysplastic syndromes is enhanced by treatment with hypomethylating agents. Leukemia (2014) 28(6):1280–8. 10.1038/leu.2013.355 24270737 PMC4032802

[6] Ishii K Barrett AJ. Novel immunotherapeutic approaches for the treatment of acute leukemia (myeloid and lymphoblastic). Ther Adv Hematol (2016) 7(1):17–39. 10.1177/2040620715616544 26834952 PMC4713888

[7] Sehgal A Whiteside TL Boyiadzis M. Programmed death-1 checkpoint blockade in acute myeloid leukemia. Expert Opin Biol Ther (2015) 15(8):1191–203. 10.1517/14712598.2015.1051028 26036819 PMC4778424

[8] Guillerey C Huntington ND Smyth MJ. Targeting natural killer cells in cancer immunotherapy. Nat Immunol (2016) 17(9):1025–36. 10.1038/ni.3518 27540992

[9] Marin R Ruiz-Cabello F Pedrinaci S Mendez R Jimenez P Geraghty DE Analysis of HLA-E expression in human tumors. Immunogenetics (2003) 54(11):767–75. 10.1007/s00251-002-0526-9 12618909

[10] Dorantes-Acosta E Pelayo R. Lineage switching in acute leukemias: a consequence of stem cell plasticity? Bone Marrow Res (2012) 2012:406796. 10.1155/2012/406796 22852088 PMC3407598

[11] Bachireddy P Burkhardt UE Rajasagi M Wu CJ. Haematological malignancies: at the forefront of immunotherapeutic innovation. Nat Rev Cancer (2015) 15(4):201–15. 10.1038/nrc3907 25786696 PMC4511812

[12] Curran KJ Pegram HJ Brentjens RJ. Chimeric antigen receptors for T cell immunotherapy: current understanding and future directions. J Gene Med (2012) 14(6):405–15. 10.1002/jgm.2604 22262649 PMC4697438

[13] Maude SL Teachey DT Porter DL Grupp SA. CD19-targeted chimeric antigen receptor T-cell therapy for acute lymphoblastic leukemia. Blood (2015) 125(26):4017–23. 10.1182/blood-2014-12-580068 25999455 PMC4481592

[14] Maude SL Frey N Shaw PA Aplenc R Barrett DM Bunin NJ Chimeric antigen receptor T cells for sustained remissions in leukemia. N Engl J Med (2014) 371(16):1507–17. 10.1056/NEJMoa1407222 25317870 PMC4267531

[15] Nagorsen D Kufer P Baeuerle PA Bargou R. Blinatumomab: a historical perspective. Pharmacol Ther (2012) 136(3):334–42. 10.1016/j.pharmthera.2012.07.013 22940266

[16] Freeman CL Gribben JG Immunotherapy in chronic lymphocytic leukaemia (CLL). Curr Hematol Malig Rep (2016) 11(1):29–36. 10.1007/s11899-015-0295-9 26857283 PMC4796351

[17] McGranahan N Swanton C. Biological and therapeutic impact of intratumor heterogeneity in cancer evolution. Cancer Cell (2015) 27(1):15–26. 10.1016/j.ccell.2014.12.001 25584892

[18] Zellmer VR Zhang S. Evolving concepts of tumor heterogeneity. Cell Biosci (2014) 4:69. 10.1186/2045-3701-4-69 25937891 PMC4417538

[19] Park M Koh KN Kim BE Im HJ Jang S Park CJ Lineage switch at relapse of childhood acute leukemia: a report of four cases. J Korean Med Sci (2011) 26(6):829–31. 10.3346/jkms.2011.26.6.829 21655072 PMC3102880

[20] Duffner U Abdel-Mageed A Younge J Tornga C Scott K Staddon J The possible perils of targeted therapy. Leukemia (2016) 30(7):1619–21. 10.1038/leu.2016.18 26859079

[21] Gardner R Wu D Cherian S Fang M Hanafi LA Finney O Acquisition of a CD19-negative myeloid phenotype allows immune escape of MLL-rearranged B-ALL from CD19 CAR-T-cell therapy. Blood (2016) 127(20):2406–10. 10.1182/blood-2015-08-665547 26907630 PMC4874221

[22] Ramos CA Savoldo B Dotti G CD19-CAR trials. Cancer J (2014) 20(2):112–8. 10.1097/PPO.0000000000000031 24667955 PMC3979594

[23] Cohen A Petsche D Grunberger T Freedman MH. Interleukin 6 induces myeloid differentiation of a human biphenotypic leukemic cell line. Leuk Res (1992) 16(8):751–60. 10.1016/0145-2126(92)90153-X 1528063

[24] Maeda K Malykhin A Teague-Weber BN Sun XH Farris AD Coggeshall KM. Interleukin-6 aborts lymphopoiesis and elevates production of myeloid cells in systemic lupus erythematosus-prone B6.Sle1. Yaa animals. Blood (2009) 113(19):4534–40. 10.1182/blood-2008-12-192559 19224760 PMC2680362

[25] Ruella M Barrett DM Kenderian SS Shestova O Hofmann TJ Perazzelli J Dual CD19 and CD123 targeting prevents antigen-loss relapses after CD19-directed immunotherapies. J Clin Invest (2016) 126(10):3814–26. 10.1172/JCI87366 27571406 PMC5096828

[26] Lapalombella R Yu B Triantafillou G Liu Q Butchar JP Lozanski G Lenalidomide down-regulates the CD20 antigen and antagonizes direct and antibody-dependent cellular cytotoxicity of rituximab on primary chronic lymphocytic leukemia cells. Blood (2008) 112(13):5180–9. 10.1182/blood-2008-01-133108 18772452 PMC2597613

[27] Bellesso M Xavier FD Costa RO Pereira J Siqueira SA Chamone DA. Disease progression after R-CHOP treatment associated with the loss of CD20 antigen expression. Rev Bras Hematol Hemoter (2011) 33(2):148–50. 10.5581/1516-8484.20110036 23284263 PMC3520640

[28] Foran JM Norton AJ Micallef IN Taussig DC Amess JA Rohatiner AZ Loss of CD20 expression following treatment with rituximab (chimaeric monoclonal anti-CD20): a retrospective cohort analysis. Br J Haematol (2001) 114(4):881–3. 10.1046/j.1365-2141.2001.03019.x 11564080

[29] Pickartz T Ringel F Wedde M Renz H Klein A von Neuhoff N Selection of B-cell chronic lymphocytic leukemia cell variants by therapy with anti-CD20 monoclonal antibody rituximab. Exp Hematol (2001) 29(12):1410–6. 10.1016/S0301-472X(01)00753-6 11750099

[30] Khong HT Restifo NP Natural selection of tumor variants in the generation of “tumor escape” phenotypes. Nat Immunol (2002) 3(11):999–1005. 10.1038/ni1102-999 12407407 PMC1508168

[31] Williams ME Densmore JJ Pawluczkowycz AW Beum PV Kennedy AD Lindorfer MA Thrice-weekly low-dose rituximab decreases CD20 loss via shaving and promotes enhanced targeting in chronic lymphocytic leukemia. J Immunol (2006) 177(10):7435–43. 10.4049/jimmunol.177.10.7435 17082663

[32] Beum PV Peek EM Lindorfer MA Beurskens FJ Engelberts PJ Parren PW Loss of CD20 and bound CD20 antibody from opsonized B cells occurs more rapidly because of trogocytosis mediated by Fc receptor-expressing effector cells than direct internalization by the B cells. J Immunol (2011) 187(6):3438–47. 10.4049/jimmunol.1101189 21841127

[33] Awan FT Lapalombella R Trotta R Butchar JP Yu B Benson DM Jr CD19 targeting of chronic lymphocytic leukemia with a novel Fc-domain-engineered monoclonal antibody. Blood (2010) 115(6):1204–13. 10.1182/blood-2009-06-229039 19965644 PMC2826232

[34] Jones JD Hamilton BJ Rigby WF. Rituximab mediates loss of CD19 on B cells in the absence of cell death. Arthritis Rheum (2012) 64(10):3111–8. 10.1002/art.34560 22674374 PMC3448861

[35] Lee S Margolin K. Cytokines in cancer immunotherapy. Cancers (Basel) (2011) 3(4):3856–93. 10.3390/cancers3043856 24213115 PMC3763400

[36] Keir ME Butte MJ Freeman GJ Sharpe AH PD-1 and its ligands in tolerance and immunity. Annu Rev Immunol (2008) 26:677–704. 10.1146/annurev.immunol.26.021607.090331 18173375 PMC10637733

[37] Hodi FS O’Day SJ McDermott DF Weber RW Sosman JA Haanen JB Improved survival with ipilimumab in patients with metastatic melanoma. N Engl J Med (2010) 363(8):711–23. 10.1056/NEJMoa1003466 20525992 PMC3549297

[38] Chester C Fritsch K Kohrt HE. Natural killer cell immunomodulation: targeting activating, inhibitory, and co-stimulatory receptor signaling for cancer immunotherapy. Front Immunol (2015) 6:601. 10.3389/fimmu.2015.00601 26697006 PMC4667030

[39] Monjazeb AM Zamora AE Grossenbacher SK Mirsoian A Sckisel GD Murphy WJ. Immunoediting and antigen loss: overcoming the achilles heel of immunotherapy with antigen non-specific therapies. Front Oncol (2013) 3:197. 10.3389/fonc.2013.00197 23898464 PMC3724213

[40] Spain L Diem S Larkin J. Management of toxicities of immune checkpoint inhibitors. Cancer Treat Rev (2016) 44:51–60. 10.1016/j.ctrv.2016.02.001 26874776

[41] Grada Z Hegde M Byrd T Shaffer DR Ghazi A Brawley VS TanCAR: a novel bispecific chimeric antigen receptor for cancer immunotherapy. Mol Ther Nucleic Acids (2013) 2:e105. 10.1038/mtna.2013.32 23839099 PMC3731887

[42] Dai H Wang Y Lu X Han W Chimeric antigen receptors modified T-cells for cancer therapy. J Natl Cancer Inst (2016) 108(7):djv439 10.1093/jnci/djv439 26819347 PMC4948566

[43] Levy EM Roberti MP Mordoh J. Natural killer cells in human cancer: from biological functions to clinical applications. J Biomed Biotechnol (2011) 2011:676198. 10.1155/2011/676198 21541191 PMC3085499

[44] Shatnyeva OM Hansen HP Reiners KS Sauer M Vyas M von Strandmann EP. DNA damage response and evasion from immunosurveillance in CLL: new options for NK cell-based immunotherapies. Front Genet (2015) 6:11. 10.3389/fgene.2015.00011 25699074 PMC4316781

[45] Deng W Gowen BG Zhang L Wang L Lau S Iannello A Antitumor immunity. A shed NKG2D ligand that promotes natural killer cell activation and tumor rejection. Science (2015) 348(6230):136–9. 10.1126/science.1258867 25745066 PMC4856222

[46] von Strandmann EP Hansen HP Reiners KS Schnell R Borchmann P Merkert S A novel bispecific protein (ULBP2-BB4) targeting the NKG2D receptor on natural killer (NK) cells and CD138 activates NK cells and has potent antitumor activity against human multiple myeloma in vitro and in vivo. Blood (2006) 107(5):1955–62. 10.1182/blood-2005-05-2177 16210338

[47] Vyas M Schneider AC Shatnyeva O Reiners KS Tawadros S Kloess S Mono- and dual-targeting triplebodies activate natural killer cells and have anti-tumor activity in vitro and in vivo against chronic lymphocytic leukemia. Oncoimmunology (2016) 5(9):e1211220. 10.1080/2162402X.2016.1211220 27757305 PMC5049355

[48] Rothe A Jachimowicz RD Borchmann S Madlener M Kessler J Reiners KS The bispecific immunoligand ULBP2-aCEA redirects natural killer cells to tumor cells and reveals potent anti-tumor activity against colon carcinoma. Int J Cancer (2014) 134(12):2829–40. 10.1002/ijc.28609 24242212

[49] Schubert I Kellner C Stein C Kugler M Schwenkert M Saul D A single-chain triplebody with specificity for CD19 and CD33 mediates effective lysis of mixed lineage leukemia cells by dual targeting. mAbs (2011) 3(1):21–30. 10.4161/mabs.3.1.14057 21081841 PMC3038008

[50] Molfetta R Quatrini L Zitti B Capuano C Galandrini R Santoni A Regulation of NKG2D expression and signaling by endocytosis. Trends Immunol (2016) 37(11):790–802. 10.1016/j.it.2016.08.015 27667711

[51] Molfetta R Quatrini L Capuano C Gasparrini F Zitti B Zingoni A c-Cbl regulates MICA- but not ULBP2-induced NKG2D down-modulation in human NK cells. Eur J Immunol (2014) 44(9):2761–70. 10.1002/eji.201444512 24846123

